# Supplementary figures and images for: DNA variants affecting the expression of numerous genes in trans have diverse mechanisms of action and evolutionary histories
Source: PLoS Genet. 2019 Nov 18;15(11):e1008375. doi: 10.1371/journal.pgen.1008375 (PMC6886874; doi:10.1371/journal.pgen.1008375)

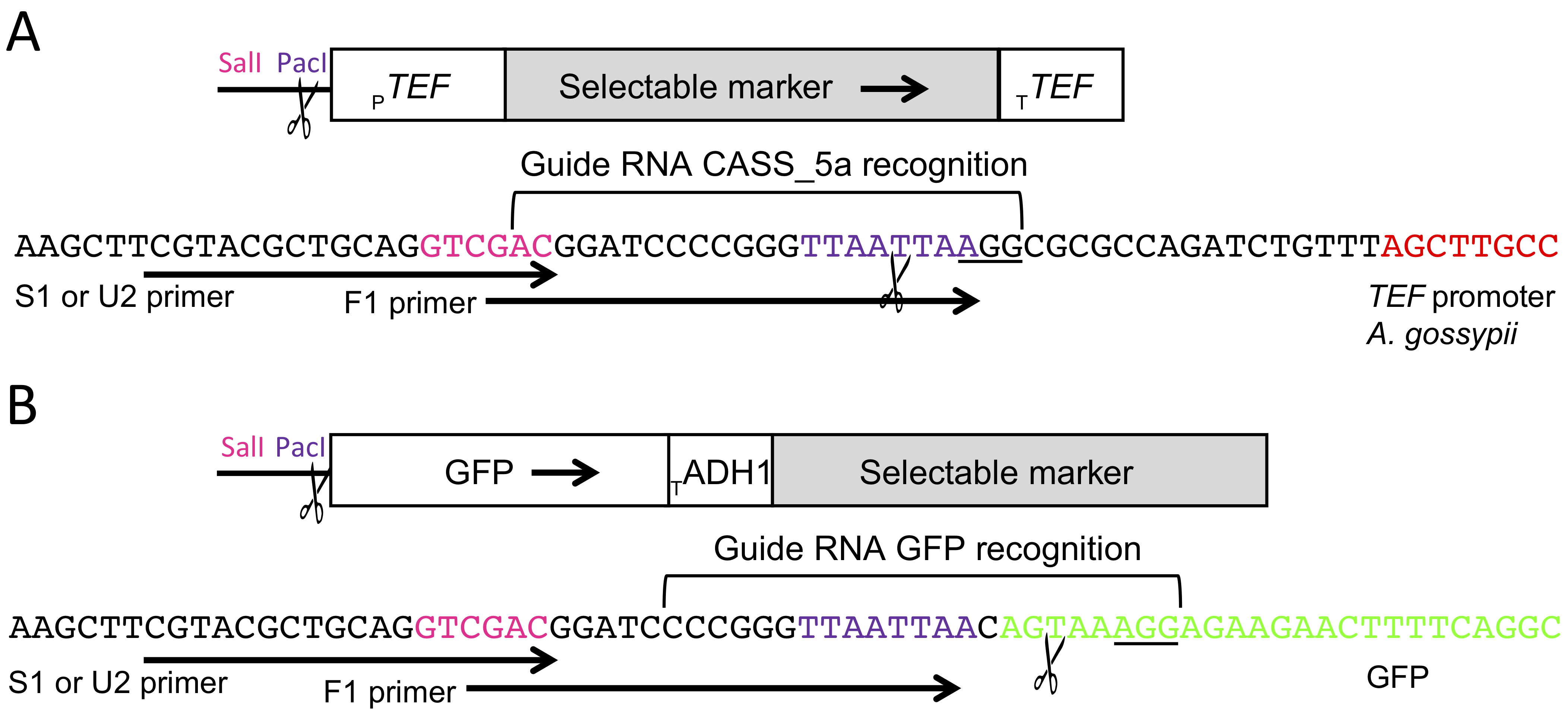

Supplement: S1 Fig — A. Schematic of a cassette typically used for gene deletions. The gCASS5a recognition sequence is marked with a bracket and the PAM site is underlined. Start of the TEF promoter sequence driving expression of the selectable marker is in red letters. B. Schematic of a cassette used for C-terminally tagging open reading frames with GFP. The location of the gGFP recognition sequence is marked with a bracket and the PAM site is underlined. The start of the GFP sequence is in neon green. The recognition sites for SalI (pink) and PacI (purple) and the Cas9 cleavage sites (scissors) are shown to allow easy comparison of the gRNA recognition sequences, which are specific to each cassette. Designated with arrows are the universal primer sequences, S1 or U2 and F1, used for amplification of common cassettes. (TIFF) [file pgen.1008375.s001.tiff]

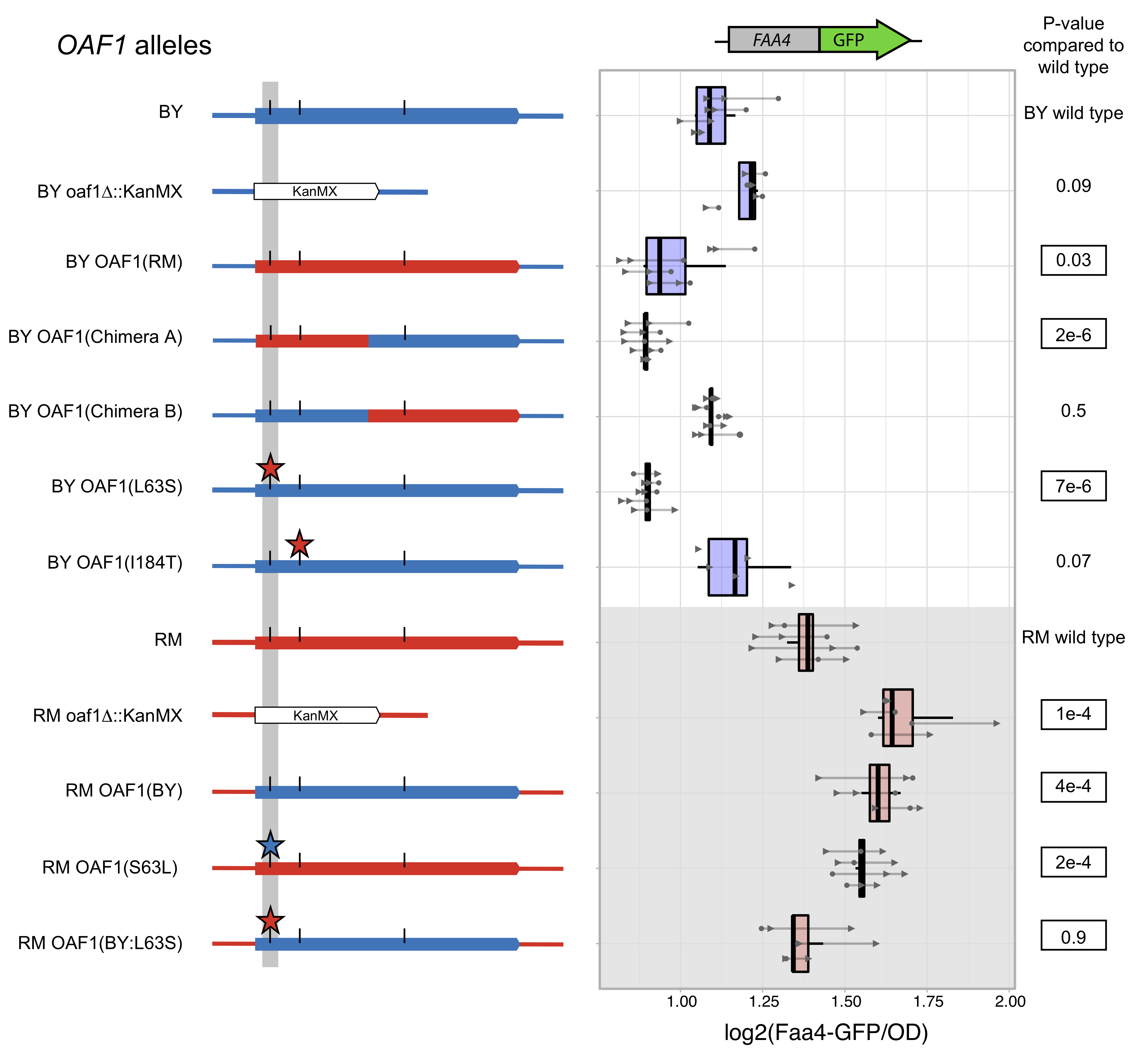

Supplement: S2 Fig — On the left are schematics of OAF1 alleles with BY sequences in blue and RM sequences in red. Missense variants are marked with a straight line. For clarity, synonymous and non-coding variants are not depicted. On the right are the corresponding Faa4-GFP fluorescence levels for each allele. P-values are for tests comparing each allele to its respective wildtype. Significant p-values are outlined. Blue boxplots indicate alleles in the BY background and red boxplots and background gray shading indicate alleles in the RM background. Lines group measurements of the same clone. Different symbols (circles, squares, etc.) denote different plate reader runs. (TIFF) [file pgen.1008375.s002.tiff]

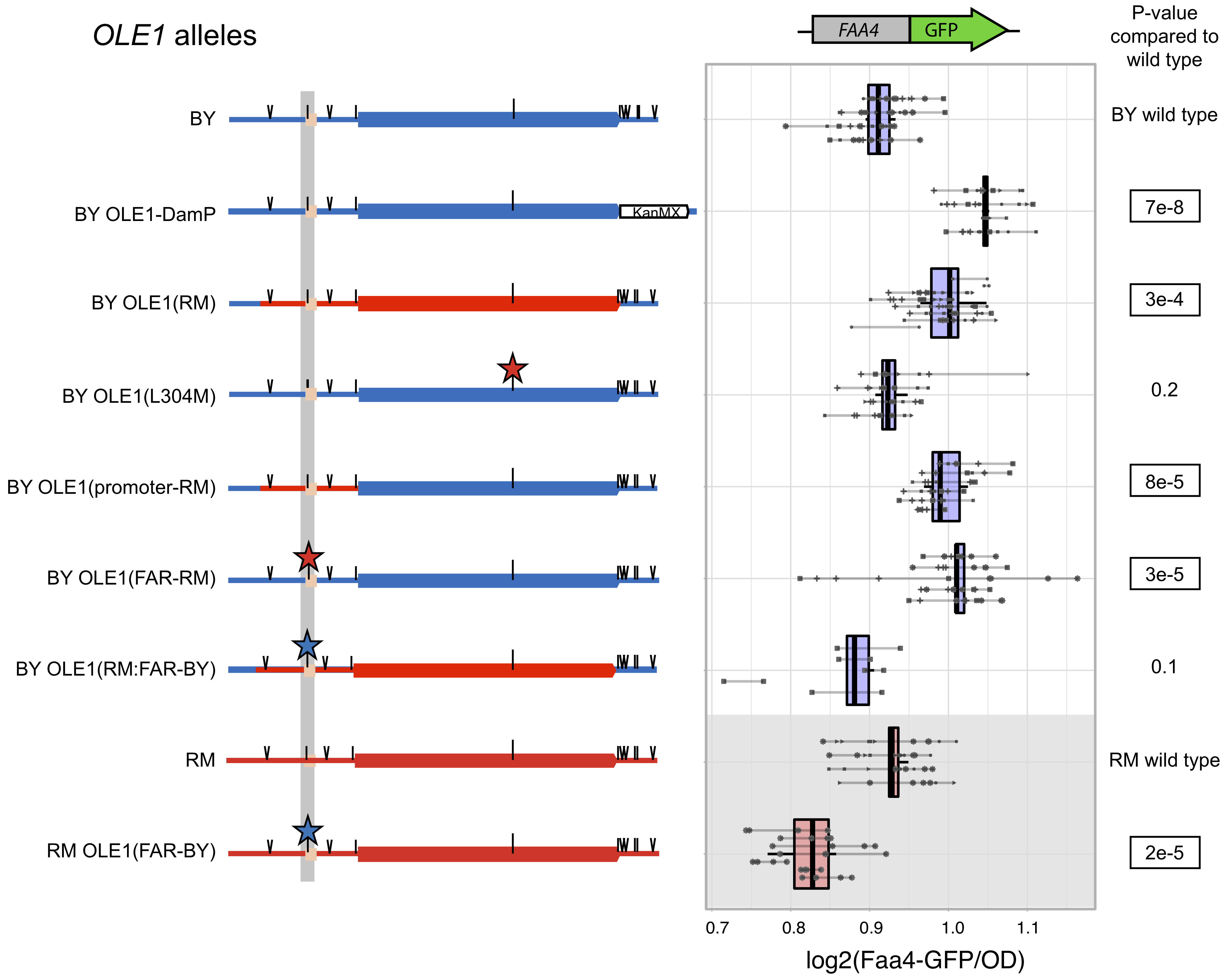

Supplement: S3 Fig — On the left are schematics of OLE1 alleles with BY sequences in blue and RM sequences in red. Only the one missense variant and none of the synonymous variants in the open reading frame are marked. Variants in the non-coding region are maked with a single line for a SNV and a two diagonal lines for INDELs. On the right are the corresponding Faa4-GFP fluorescence levels for each allele. P-values are for tests comparing each allele to its respective wildtype. Significant p-values are outlined. Blue boxplots indicate alleles in the BY background and red boxplots and background gray shading indicate alleles in the RM background. Lines group measurements of the same clone. Different symbols (circles, squares, etc.) denote different plate reader runs. (TIFF) [file pgen.1008375.s003.tiff]

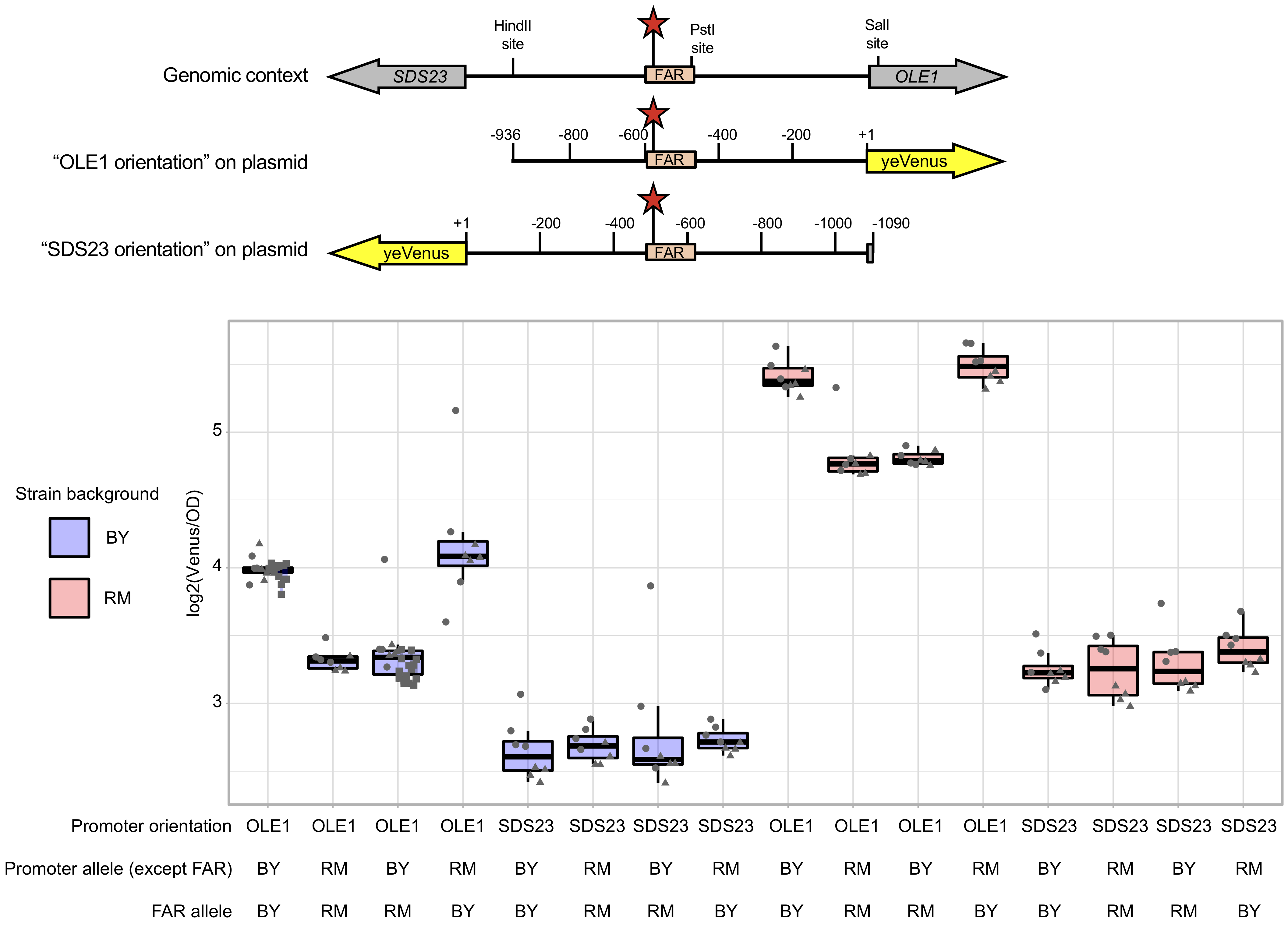

Supplement: S4 Fig — On the top are schematics of the SDS23/OLE1 locus and the two orientations of the yeVenus reporter constructs. The bottom panel shows yeVenus fluorescence levels for the indicated yeVenus reporter constructs. Blue boxplots indicate alleles in the BY background and red boxplots indicate alleles in the RM background. Lines group measurements of the same clone. Different symbols (circles, squares, etc.) denote different plate reader runs. (TIFF) [file pgen.1008375.s004.tiff]

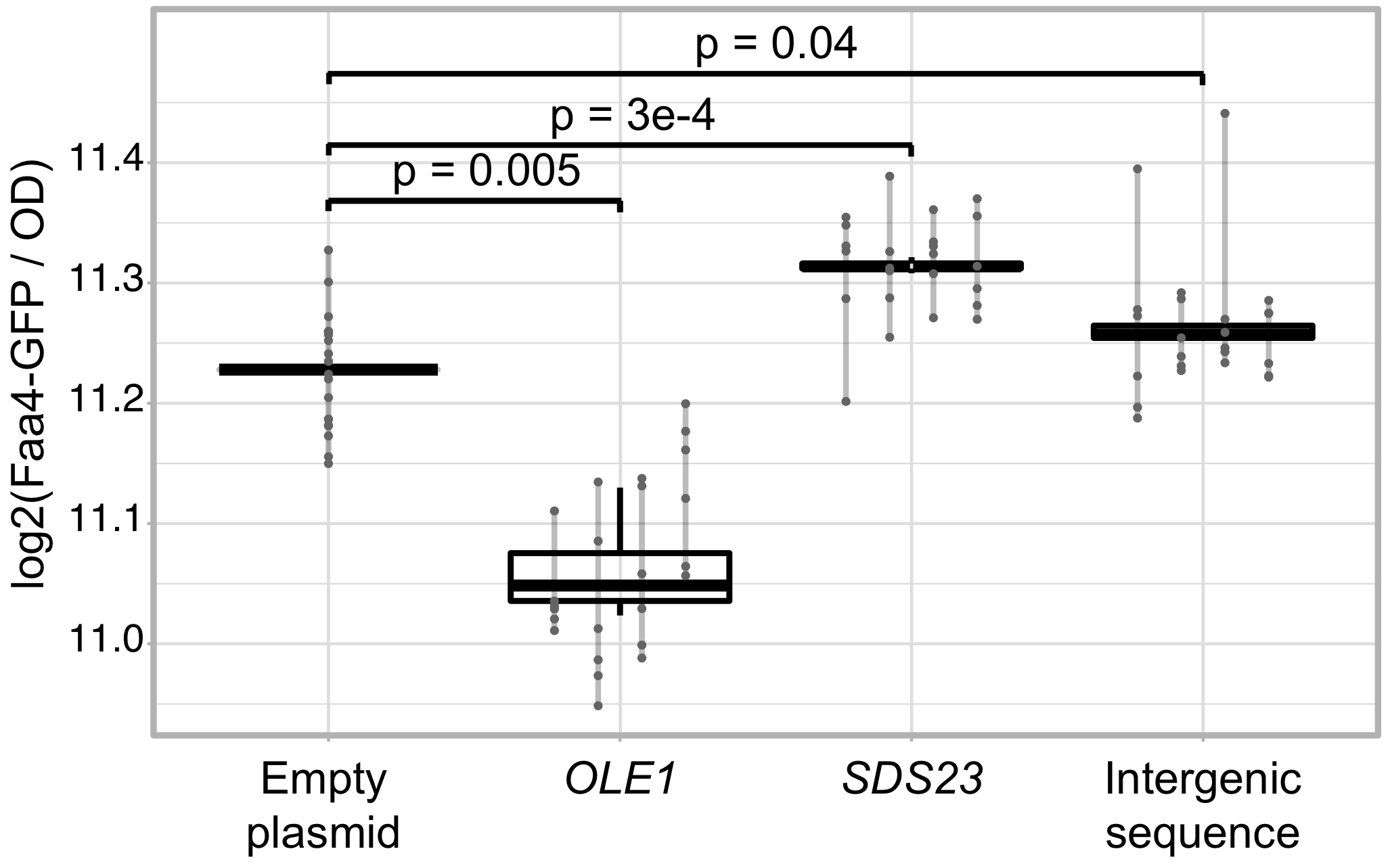

Supplement: S5 Fig — Faa4-GFP fluorescence levels of strains transformed with a LEU2-CEN plasmid containing the indicated sequence. Lines group measurements of the same clone. (TIFF) [file pgen.1008375.s005.tiff]

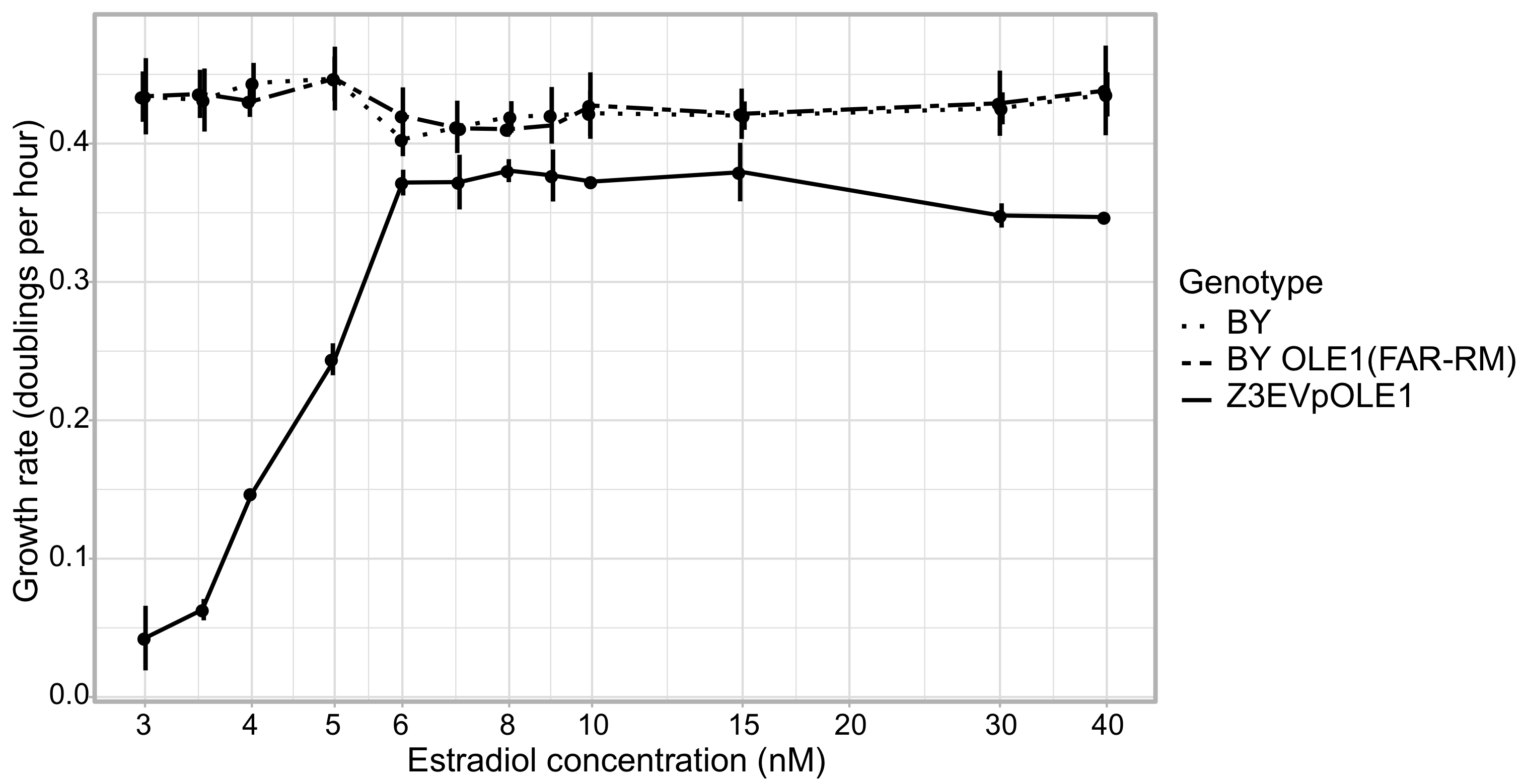

Supplement: S6 Fig — Error bars show standard deviations. (TIFF) [file pgen.1008375.s006.tiff]

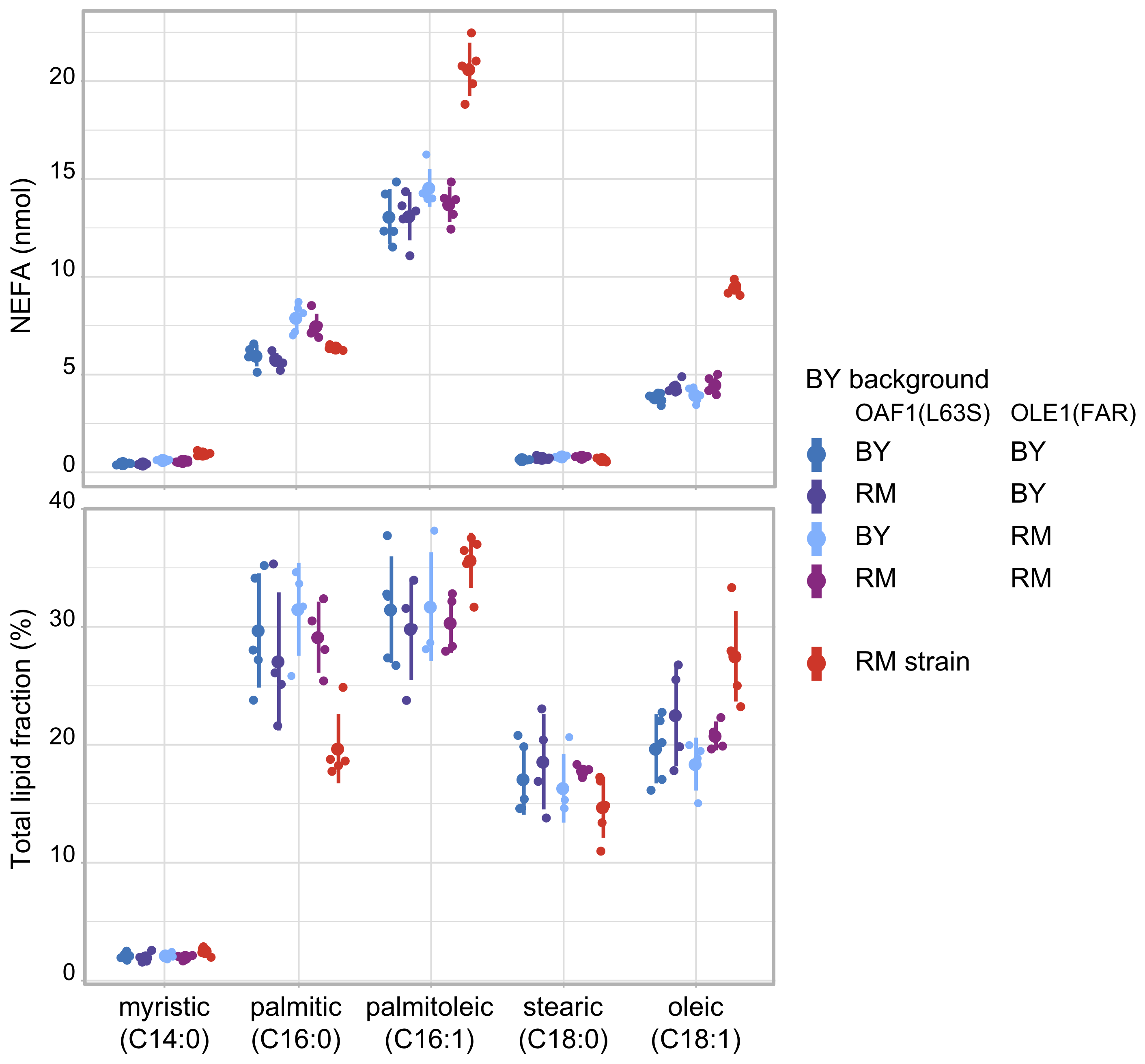

Supplement: S7 Fig — All individual measurements are shown. For each genotype, the figure shows values for each replicate (smaller points) along with the mean (larger points) and standard deviation (vertical lines). (TIFF) [file pgen.1008375.s007.tiff]
