# Supplementary material for: DNA variants affecting the expression of numerous genes in trans have diverse mechanisms of action and evolutionary histories
Source: PLoS Genet. 2019 Nov 18;15(11):e1008375. doi: 10.1371/journal.pgen.1008375 (PMC6886874; doi:10.1371/journal.pgen.1008375)
Supplement: Attachment — Submitted filename: ResponseToReviewers_191022.pdf [file pgen.1008375.s019.pdf]

Response to Reviewers

Reviewer comments are shown in Courier font.

Our responses are in Times New Roman.

Reviewer #1

In the manuscript, "DNA variants affecting the expression of numerous genes in trans have diverse mechanisms of action and evolutionary histories", the authors provide a thorough and interesting investigation of three trans eQTLs implicated as hotspots for expression change, and in the process present a technique for allele swaps that will be of broad interest to the yeast community. The manuscript is written clearly and compellingly, and addresses a broad and important question. It ranges from detailed functional analysis to an investigation of the phylogenetic distribution of variants. As I read the manuscript, every technical and statistical question that arose ended up being addressed. In fact, I commend the authors on the detailed methods, the rationale for the technical and statistical choices, and the inclusion of all the results and data. With regard to the journal's criteria: originality, broad interest, solid methodology, and support for conclusions, the manuscript hits all those points. As such, I only have general comments and specific minor suggestions.

We thank the reviewer for this positive assessment.

First, with regard to the main question of manuscript, which is determining the molecular basis of trans eQTLs, there is no a priori reason to assume that one type of molecular mechanism will be more common than another (i.e., I don't think it is a surprise that trans-acting eQTLs can be conserved or not, regulatory vs. coding, functionally diverse proteins, etc.). In fact, given the delicate dance that goes on within cells that sense and respond to their internal and external environments, it is not surprising that all mechanisms can be found. Using descriptors like "remarkable diversity" seems a little too much. That being said, it is important to demonstrate that all types of mechanisms are possible with solid examples and this study does that incredibly well.

We appreciate the reviewer's comment of the importance of demonstrating how different causal variants can be. Following the suggestion, we have slightly rewritten the abstract to remove the word "remarkable" and the discussion to remove the word "striking".

Second, I think that it is quite interesting that all of the derived alleles seem to be in the direction of null alleles, given the current public conversation about loss-of-function mutations (https://onlinelibrary.wiley.com/doi/full/10.1111/evo.13710). While it may out of the scope of this manuscript, a sentence or two in the

discussion discussing the relevance of these findings would put this observation in a broader context.

We thank the reviewer for the link to the insightful review. We agree that it is interesting that all our variants have effects in the same direction as loss-of-function mutations. However, we do feel that further discussion of this point from the perspective of evolutionary adaptation would be too speculative given the data. Specifically, we do not know if any of our three variants have adaptive or deleterious consequences. We have therefore chosen to keep our discussion of these variants as is.

Third, I strongly suggest the authors give a brief description of the study that this paper follows up on. For example: How many eQTLs were found in this study (what was the punchline)? What media was used (this matters later on when glucose level in the medium is tested)? I understand the three loci chosen from that study for dissection were chosen because they were mapped to a small genomic region (which makes sense), but a little bit of an overview of the previous work would be helpful.

As suggested, we have added additional information about the previous eQTL dataset. In the introduction, we now present the total number of eQTLs (>36,000) and the "punchlines" that 1) these eQTLs account for the majority of gene expression heritability in the BY/RM cross and 2) that essentially all *trans* eQTLs co-occur at hotspots. We have added the medium used (YNB with 2% glucose) to the Results section describing the *RGT2* GxE experiment. We have also provided additional information about why we selected the three loci we dissected here – briefly, these decisions were based on the hotspots being relatively narrow and having available abundantly expressed genes strongly affected by the locus.

Finally, with regard to the RGT2 story, how do the levels of glucose tested compare to anything the yeast might see in the environment? Are they comparable?

This is an excellent question to which we don't have a good answer. The levels of glucose in the environment are likely to fluctuate substantially more than they do in the laboratory. For example, during wine fermentation glucose concentrations can reach up to 20%, while some environments may contain no glucose at all.

Is it possible to test 0.5% and 0.1%?

We did in fact conduct preliminary tests with 0.5% but not 0.1% glucose. At 0.5%, the effect of the V539I variant was somewhat *smaller* than at 1% and 2%, and *HXT1*-GFP expression was lower than at 1%. At lower glucose concentrations, the high affinity glucose receptor, Snf3 is the predominate glucose sensor. Due to a possible role of both Rgt2 and Snf3 at glucose concentrations below 1% we chose not to include these results in the paper and because we feel they do not add substantially to the overall conclusion that the effect of this variant is highly dependent on glucose concentration in the medium.

What level was in the medium of the original study in which this locus was implicated?

2%. This is now noted in the *RGT2* GxE section. We also added an asterisk to the 2% glucose concentration and a comment in the legend to Figure 3.

Since the paper goes on to look at the distribution of these alleles, it is worth thinking about why we see the distribution we do and what the strains in the phylogenetic tree are likely to experience.

This is another excellent point, but we do not feel that we have the data to meaningfully address it. Given the high dependence of the V539I effect on glucose combined with the idea that glucose is probably not constant in natural yeast environments, it is difficult to relate the position of this variant in the tree to its function.

Minor:
It is a bit hard to see the shapes of the points in Figure 5C. There also seems to be more variation in these data than the others. Can the authors comment on why they think that is in the manuscript (I suspect it is simply because the effect of the SNV is smaller).

We have changed the sizing of the points in Figure 5B (there is no Figure 5C, so we assume the reviewer is referring to 5B) and Figures 3, 4, 7 and S04. We agree that the apparent additional variation for the FAR variant is likely to be a function of a smaller effect of this variant, but aren't sure what stating this in the manuscript would add.

In figure 4C, it took a bit for me to comprehend the results. This is in part because in figure 2, the allele swaps essentially recapitulate the two different wildtype levels and that is what the reader expects to see (the comparable figures for the other alleles are in the Supplemental Material). However, in figure 4, swapping the causal variant does not lead to the wt level of the other background. The authors might consider putting small arrows next to the RM and BY labels on the x-axis where the allele is specified. A little up arrow next to BY and down arrow next to RM would remind the reader of the expected direction of the effect of the allele swap. But this is a style choice for the authors.

While we agree that this inconsistency between figures is perhaps not ideal, we feel it is better to show the data as is, opposed to, say, perform another normalization that would set the two wildtypes to the same value. Currently figure 4C does convey the information that RM has higher *FAA4* levels. Interestingly this effect is clearly not due to RM's *OAF1* allele, because the BY allele increases *FAA4* levels even further – as such it is transgressive compared to the strain difference.

To help readers interpret the figures, we have added arrows indicating the expected direction of effect to Figures 2, 4, and 5.

`Line 172: “There” should be “The”`

Fixed; thanks for spotting this.

Reviewer #2

This manuscript describes fine mapping of previously identified eQTL hotspots down to the causative polymorphisms. The authors use a two-step "CRISPR-SWAP" strategy that can be adapted to swap essential genes. The authors validate the approach on three candidate hotspot genes, using GFP fusions to genes affected by each hotspot. The causative variants included two missense variations and one cis variant that affected a DNA-binding site. The authors explored both GxE and epistatic interactions across hotspots and found evidence for both. The authors performed RNA-seq on causative alleles to show that they affected more than just the single reporters tested. Beyond gene expression, the authors show that two hotspots that affect genes involved in fatty acid metabolism indeed have allele-specific effects on cellular lipid profiles. Overall, this paper is well written, the data is generally well presented, and the interpretations are mostly sound. I do offer some critiques for the authors to address. (And while I have quite a few comments, I should stress that these are mostly asking to clarify for the reader).

We thank the reviewer for this favorable assessment and thorough comments below.

1) While I appreciate the advantages of the authors' CRISPR-SWAP method, I think it's fair to point out that CRISPR has been used before for allele swaps in yeast (PMID 29114020)

We have added a reference to this paper as well as to another paper using two different guide RNAs flanking a region to the Discussion.

2) The authors claim that CRISPR-SWAP is 2-step, but is that true for the essential genes? Were the Kan and Hyg markers introduced simultaneously into the OLE1 region?

We agree. Indeed, our intention in using the term "two-step" was mainly to distinguish our approach from "single-step" CRISPR engineering, in which the given locus is targeted with a specific gRNA. We have removed all instance of "two-step" from the paper, other than when we refer to other methods in the Discussion.

3) The authors claim that they obtained unexpected chimeric alleles for the OLE1 swap, which they interpret as being due to homology between the OLE1 repair template and the intervening sequence between the cassettes. This should be true of any swap of essential regions, right? I think a recommendation of sequencing of allele swaps for essential regions is warranted.

We have added this recommendation to the Methods section.

4) Line 1413: "and" instead of "are," unless I am not understanding.

Correct. This is now fixed.

5) For the completeness of the literature review of hotspots, Oaf1 was at least hypothesized to be causative for an eQTL hotpsot in PMID 19223586.

We have added this reference to the OAF1 results section.

6) Line 348: Instead of calling the CEN plasmids single copy, it is probably better to call them low copy (see PMID 23107142).

We agree and have changed “single” to “low”.

7) I’m not sure what to make of for SDS23 overexpression having an effect in Fig S5. At first I thought that maybe having extra copies of the FAR sequence could affect TF binding at other sites, but the intergenic sequence doesn’t have that effect. Based on SDS23 function, do the authors have any thoughts?

We do not have a good explanation for this observation. Indeed, we included the intergenic sequence without an ORF specifically to control for any effects an extra copy of the promoter elements may have had. *SDS23* is not a well-studied gene, and appears to play a role in the cell cycle and yeast budding. We do not see an interpretable functional link that would relate it to *FAA4* expression. The fact that its level does seem to be able to influence *FAA4* expression is certainly tantalizing, but we feel that an exploration of this effect is outside of the scope of this manuscript.

8) Fig6: what do the lines above and below each point denote (e.g. standard deviation)?

Thanks for catching this omission: these are indeed standard deviations. This has been added to the figure legend.

9) For Figure S6, what does the y-axis denote? Growth rate is usually in the units time^-1, but negative values would not make sense then.

These values were log-transformed growth rates, which in turn were in units of population doublings per hour. The rates were estimated using the growthcurver package in R, which in the manual defines growth rates as the “intrinsic growth rate of the population, r, is the growth rate that would occur if there were no restrictions imposed on total population size”. For example, a doubling time of 2 hours corresponded to a value of -0.69.

We agree that the log-transformed display was confusing and have changed the y-axis to a more conventional doublings per hour.

10) Line 368: The logic of why estradiol-inducible OLE1 requires higher expression than OLE1 under the native promoter is not clear to me. It seems far-fetched that the inability to tune OLE1 at lower expression levels would have such a dramatic effect on growth, while expressing much higher levels is apparently fine. Not that I can think of a better explanation. Note: this assumes that the effect really is dramatic given my inability to grok the y-axis for Fig S6.

Please see our explanation of the y-axis in figure S6 above; it is now displayed in doublings per hour.

We do not know why the Z3EV strain does not reach the full growth rate of the strain with its native promoter. The fluorescent tags at *OLE1* and *FAA4* are the same in all strains in this experiment. The differences in growth rate must therefore be due to the replaced *OLE1* promoter (as we speculated in the paper) and/or due to the cost of making the synthetic Z3EV transcription factor. Given we cannot currently do better than speculate, we have chosen to no longer highlight this observation in the text. Figure S6 remains available for interested readers.

11) For Figure 7A, I'm not sure that the blue/red color scale works. For the other figures, blue/red denotes BY vs. RM genetic background for allele swaps, which is what I thought this denoted at first. Of course, this makes no sense when looking at segregants for the eQTL mapping, which left me a bit confused until I noticed in the key that the colors denote OAF1 allele. It may just be me, but I think that other readers may get acclimated to the blue/red BY/RM scale.

We agree, and have changed the color scheme in Figure 7 to be consistent with the other figures. Specifically, the panel showing the segregants now has box plots colored purple to indicate that the segregants plotted here do not have a uniform background genotype but rather are a random combination of BY (blue) and RM (red) alleles. The other two panels showing our engineered strains now have blue boxplots, consistent with the BY background used in these strains.

12) While perhaps not statistically significant, variation in OLE1 FAR does seem to affect lipid composition with a similar direction for both the RM and BY OAF1 variants. Based on the directionality, this seems important enough to at least mention.

We assume that this comment relates to Figure 8, which appears to show that summed C18 lipids are slightly lower with the RM FAR allele than with the BY allele. However, examination of the p-values (Supplementary Table S3) shows that this comparison is never close to significance: the lowest p-value for a FAR effect is 0.4, for the palmitic acid (C16:0) fraction. Given these weak p-values we prefer not to highlight any potential effects of the FAR variant on lipid composition. Of course, all lipid and fatty acid measurements (Supplementary Table S2) and the results of our statistical analyses (Supplementary Table S3) are available with the paper, such that interested readers can draw their own conclusions.

```
13) Line 433: The logic here does not make sense to me. How can
reduced substrate consumption not also result in reduced product? I
think there may be something going on at the total lipid level to
compensate. Instead of just the NEFAs, what happens if you show the
total sat/unsat for the total lipids?
```

We agree with the reviewer that processes other than the reaction catalyzed by Ole1 are likely responsible for this observed compensation of unsaturated NEFAs. Our statement in the paper was unclear because it may have seemed to imply that the FAR allele *itself* performs this buffering. We have rewritten these statements for clarity.

As suggested, we also looked at the summed saturated and unsaturated fraction of the total lipids. As shown in the figure below as well as in Supplementary Table S3, there is no difference in these measures as a function of either the *OAF1* or the FAR allele.

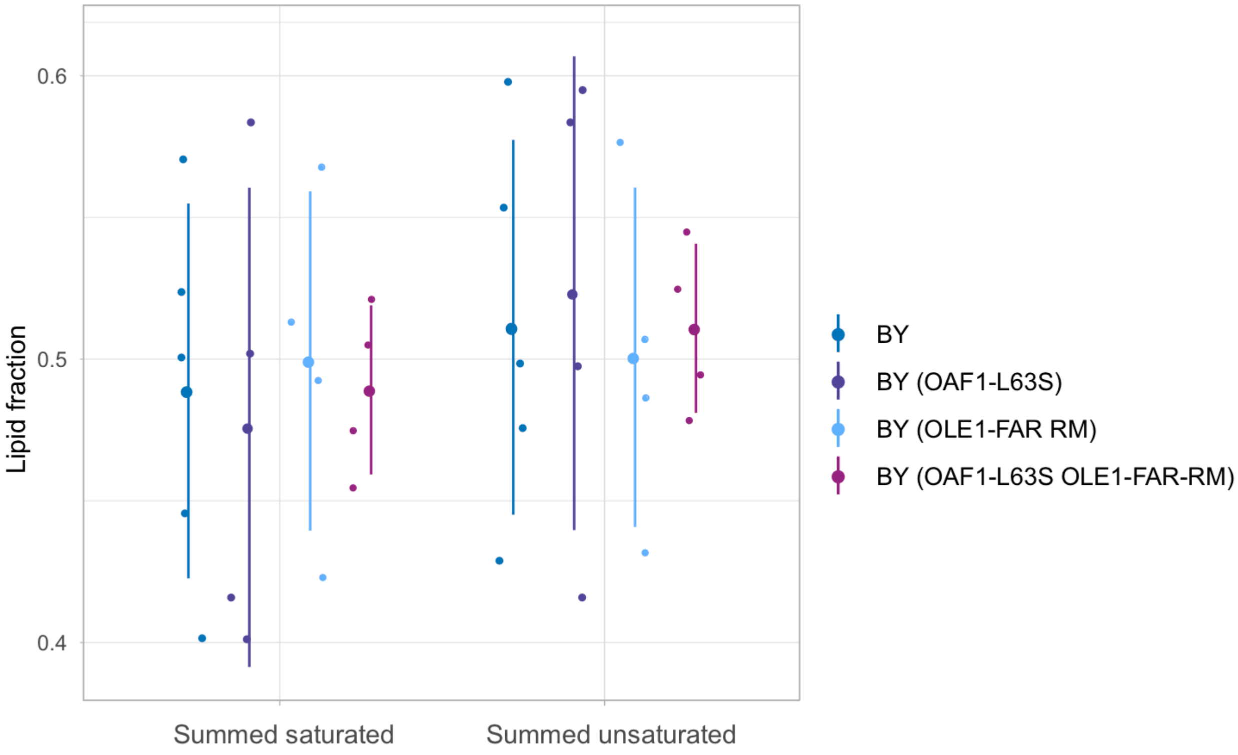


```
14) For overlap of the hotspot genes with the DE transcripts for each
variant, it would be useful to also look at the percent overlap of
differentially expressed genes with the hotspot-affected genes and
Fisher's exact test for significance.
```

We have computed the suggested statistics. Briefly, we observe highly significant Fisher's exact tests for all hotspots, irrespective of whether differential expression is defined at an FDR <10% or a nominal p-value of 5%. We have added the results from the Fisher's exact test to Table 2. We opted to show only the results for all genes in the table; i.e. without requiring hotspot effects

to exceed a certain value. The strength of association between hotspot effect and differential expression increases when requiring the hotspot effect to be at least of a certain magnitude, in line with the expectation that our RNASeq experiment is better able to detect large effects.

To accommodate this additional column, we have removed the column showing the correlation between hotspot effect and differential expression *for all genes* from the table; this information was redundant with and remains available in Figure 9.

`15) For Figure 9, it's not clear to me what hotspot effect means. Is this essentially the difference in log2 expression when you sort on the segregants at that genotype? The methods of this paper point to the Albert et al. eLife paper, but I couldn't really parse what "hotspot effect" meant from that paper.`

The "hotspot effects" in Figure 9 are regression coefficients obtained from regressing the expression values in the segregants against the genotype at the hotspot marker in our earlier eLife paper. That regression had been computed with lasso-based regularization to avoid overfitting of effects when regressing every gene against 102 hotspot positions.

Here, we chose these "hotspot effects" for an additional practical reason: they are available as a precomputed matrix ("Source Data 9") of hotspot effects in our earlier paper. The Methods do already contain an explicit reference to that source data.

Because these hotspot coefficients are not log2 fold changes, we calculated their correlation with differential expression in our new data (which *is* expressed as log2 fold change) as a nonparametric Spearman's rank correlation rather than a parametric Pearson correlation.

We have added information on the "hotspot effects" to the legends of Figure 9 and Table 2.

`16) For the Discussion, I would have liked to see more on the role of both Oaf1 and Ole1 on regulating FAA4 expression. Based on the function of Oaf1 in fatty acid metabolism, does it make sense that FAA4 expression would increase as Oaf1 activity decreases? Is there an Oaf1 site in the FAA4 promoter?`

Although we cannot rule out a direct binding of Oaf1 to the *FAA4* promoter, the *FAA4* promoter does not contain a consensus Oaf1 binding site, also known as an oleate response element (ORE) (PMID:18671944) nor has it been shown to be bound by Oaf1 using ChiP (PMID:30073202).

`Likewise for reduced Ole1 activity—would you expect compensatory FAA4 expression?`

See below for our answer to comment 19 from the same reviewer on how Ole1 activity might affect *FAA4* expression via Mga2. Given fatty acid metabolism is highly complex and characterized by feedback and multiple layers of regulation, we prefer not to speculate further about why and exactly how *FAA4* would respond to reduced *OLE1* activity.

`Also, does FAA4 have a FAR site (and is it known what TF binds to that site)?`

The 111-bp fatty acid-regulated element (FAR) in the *OLE1* promoter is somewhat unusual among regulatory “motifs” in that it has been defined solely in the *OLE1* promoter as a region necessary for repression of *OLE1* in the presence of unsaturated fatty acids. As such, *FAA4* does not have a FAR site.

`17) Lines 569-570: yeast are rather gene dense at the genome level, so the predominance of coding to non-coding variation may be a function of target size.`

This is a good point, and we have added a brief reference to this fact to the discussion (line 655).

`18) Line 574-576: while I think it’s most likely that enzymes exert hotspot effects via alterations of metabolites, I would also bet that they are more likely to have moonlighting functions than expected by chance. In the case of Ole1, I think the jury is out until mechanism is elucidated.`

It is true that enzymes (or any other protein) could have functions other than the ones they are commonly known for. It this for this reason that we stated “***most*** enzymes do not directly regulate gene expression” in this section (emphasis added). In our view, it remains true that enzymes are more likely to cause gene expression change indirectly compared to, say, transcription factors. We have therefore chosen not to change the text.

`19) Lines 580-582: the authors speculate that the OLE1 FAR allele could exert its hotspot effects via Mga2. Are genes affected by the OLE1 hotspot more likely to Mga2 targets (by ChIP or expression data)?`

Another excellent question, which we had explored during our analyses. Specifically, we correlated the gene expression changes caused by the FAR variant with those caused by overexpression of the active domain of the Mga2 protein, as published in PMID:28323063. Interestingly, we did find a highly significant correlation (Spearman’s rho = 0.16 across all expressed genes, p=1e-31). This correlation with the active Mga2 domain was stronger than that with expression changes after overexpressing the active Spt23 domain from the same reference (rho = 0.05, p=0.0002). These analyses informed our speculation that the consequences of the FAR variant might ultimately be affected by Mga2 level/or function. Because they remain somewhat inconclusive, we had decided to leave these results out of the (already long) paper.

For this revision, we have added an additional reference (PMID:16777852) to the discussion to highlight several individual known Mga2 target genes that are also strong targets of this hotspot and the FAR variant in our new expression data.

Reviewer #3

This manuscript describes fine-mapping of the causal nucleotides for expression differences between Saccharomyces cerevisiae isolates BY and RM located in previously defined eQTL "hot spots" for trans expression variation. To do so, the authors introduce a CRISPR-Cas9 based method, "CRISPR-Swap", analogous to traditional marker-assisted homologous recombination but using CRISPR-directed cutting against an integrated selection marker to generate a double-strand break that can then be repaired with a library of sequence variants. For each of three eQTL hot spots, the experiment reported here maps a causal variant affecting one focal gene linked to the eQTL in trans using a fluorescent reporter for the focal gene. Identifying the frequency of those causal variants in context of S. cerevisiae population variation is a nice additional flourish.

The problem of understanding trans contributions to gene expression variation is a hard one. Because the problem is so hard, there are some features of this manuscript that are unsatisfying, despite what I believe is ultimately a well-rounded and useful contribution to describing the genetic basis of expression variation. The authors tackle the problem of defining the causal nucleotides for gene expression differences within three eQTL hot spots, but analyze the consequences of sequence differences on a single focal trans gene in each case. (RNA sequencing after flipping of the fine-mapped causal nucleotide provides support for the conclusion that the fine-mapped variants alter expression for more than just the mapped gene, but with limited power.) Further, fine-mapping is performed across a subset of SNPs within the eQTL interval based on their overlap with candidate genes. This approach provides useful examples of how single nucleotides can influence trans expression differences. It cannot fully address whether the single variant explains all the trans effects mapped to eQTL hotspots or whether multiple SNPs colocalized in the same region might contribute to trans effects on different genes. Other groups have published methods that might provide the necessary firepower to do a higher throughput analysis of more variants to answer these questions (in some cases working on the exact same genetic cross), but applying those methods to comprehensively fine-mapping trans expression differences remains a non-trivial undertaking. This manuscript fills that gap by expanding the number of documented SNPs impacting expression in trans, and does so with a nice attention to the potential functional consequences, environmental dependence, genetic interactions, and population context of the SNPs mapped. The range of differences between even these three examples offer useful hypotheses moving forward for how segregating genetic variation can have cascading and interacting impacts on gene expression and cell physiology. As a result, the work provides a useful illustration of the complexity of understanding trans effects on gene expression variation.

We thank Dr. Hodgins-Davis for her detailed and spot-on summary of the strengths as well as weaknesses of our manuscript. We especially agree with the observation that the variants we

identified need not be the only ones in their respective regions. Indeed, we have provided evidence that at the *RGT2* locus, an additional variant likely affects multiple *ADE* genes.

Questions to address:

1. In the general the manuscript is well written and easy to follow. In the introduction, it would be helpful if the authors could (briefly) make the case for what is gained by knowing the causal nucleotide within an eQTL. (An additional sentence or so on this point would do it, but given the history of critique of the QTN program, why do it here?)

We have added a sentence motivating why, in our view, dissecting causal nucleotides in eQTLs is a worthy pursuit (lines 72 – 76).

2. Please explain the choice of these three loci for fine-mapping over other eQTL hot spots identified in prior work.

We have expanded our explanation of why we chose the three hotspots for this manuscript (lines starting 187). Briefly, this choice was based on a series of considerations that included 1) a narrow hotspot based on prior mapping results, 2) the availability of a good candidate gene given the enriched functions of the targets of the hotspot and on prior literature, 3) the availability of a good "phenotyper" gene that was strongly affected by the locus, had functional coherence with the enriched functions of the hotspot's target genes, and had high enough expression that when tagged with GFP could be detected in the plate reader.

These criteria resulted in several hotspots we could have chosen for further dissection. We selected the three in the paper with an eye towards wanting to demonstrate that causal trans nucleotides can be diverse in terms of the genes they reside in. Further, we selected the *OLE1* and *RGT2* hotspots in part because they had local eQTLs at the likely causal genes. We did this because prior work had found few instances of causal hotspot nucleotides in noncoding regions in yeast crosses, and we wanted to increase our chances of finding more examples (or failing at doing so, which would also have been interesting). Finally, *OAF1* is quite simply the "first" hotspot in the sense that it is located at the left-most end of chromosome I, and as such caught our eye when examining hotspot lists.

Together, this selection scheme may of course result in a biased set of causal variants, as would have been true for any set of "only" three causal variants compared to a hypothetical comprehensive, genome-wide dissection. Our intention was not to draw conclusions about the typical nature of causal variants, but instead to provide carefully worked examples of diverse mechanisms that can underlie trans-acting hotspots.

3. The GFP screen taking into account the growth curve of the strain is elegant. I miss a little more experimental detail about how this screen was executed, including technical information about how

replicates were compared within vs between independent runs, whether position or edge effects across a plate were taken into account, etc.

Edge effects (or any other position effect within a plate) were not explicitly considered in the statistical analyses. Because every strain was present in multiple different wells distributed across different sectors of our measurement plates and usually also across different plate runs, we believe that ignoring edge effects is appropriate. We have added this information to the Methods section (lines starting 840 and 1002).

We are not entirely sure what other information to add about the measurements and their analysis, given that in the Methods we had already provided 1) detailed descriptions of how we grew strains prior to loading as well as in the plate reader, 2) our detailed measurement settings, and 3) a dedicated description of how biological (different clones from one transformation) and technical (different wells from the same clone) replicates were handled in the analyses (See section "Statistical analyses of plate reader data"). Note that we also provided all plate reader data including plate layouts, as well as all analysis code.

4.Is RGT2 itself plastic to glucose level?

We have not measured the mRNA or protein levels of *RGT2* in different glucose concentrations. The expression level of an RGT2::lacZ reporter was the same in 0.1% glucose and 4% glucose, suggesting that Rgt2 expression is constitutive (PMID: 8901598).

5.Minor concerns:
a. The blue and red fill for boxplots are challenging to distinguish in areas where the confidence intervals are tight. In places like Fig 3, I suggest adding labels (perhaps in a row at the top of the plot?) to distinguish alleles.

We agree that the blue and red fill boxplots are challenging to distinguish, especially in Figure 3. We have modified Figure 3 with BY and RM background labels along the x-axis in addition to the gray shading behind strains in the RM background.

b. The symbols that represent different plate reader runs are also very challenging to make out at current sizing. I understand the desire to minimize the visual impact of this technical information, but if provided, the size of the symbol should be large enough to read.

We have increased the size of these symbols.

c. Is there a reason for the choice to display hot spot 95% confidence intervals for RGT2 and 90% confidence intervals elsewhere?

This is an astute observation. For *OAF1* and *OLE1*, the 90% and 95% confidence intervals happened to include the exact same respective sets of markers, such that in the figures, the 90% intervals fully occlude the 95% interval. We have added this explanation to the legends of figures 4 and 5.
